# Implementation of TREC/KREC Newborn Screening in a High-Birth-Rate Population: A Pilot Study of 5000 Neonates in South Kazakhstan

**DOI:** 10.3390/ijns12030051

**Published:** 2026-07-07

**Authors:** Gulzada Abdushukurova, Alken Auyelova, Banu Kadyrbayeva, Ardak Ayazbekov, Dina Mussayeva, Ainash Oshibayeva, Kumissay Babayeva, Liliya Khairullina, Karlygash Sadykova, Gulnaz Nuskabaeva

**Affiliations:** 1 Department of Therapy, Khoja Akhmet Yassawi International Kazakh-Turkish University, Shymkent Institute of Postgraduate Studies, Shymkent 160021, Kazakhstan; gulzadabdushukurova@gmail.com (G.A.); banu.kadyrbayeva@gmail.com (B.K.); 2 “San-Med Service” Allergy Center, Shymkent 160000, Kazakhstan; dr.dina-t@mail.ru (D.M.); khairullinaliliya7@gmail.com (L.K.); 3 Faculty of Medicine, Khoja Akhmet Yassawi International Kazakh-Turkish University, Turkestan 161200, Kazakhstan; ardakayazbekov966@gmail.com (A.A.); ainash.oshibayeva@ayu.edu.kz (A.O.); 4 Department of Special Clinic Subjects, Khoja Akhmet Yassawi International Kazakh-Turkish University, Turkestan 161200, Kazakhstan; altinay.babayeva@ayu.edu.kz (K.B.); karlygash.sadykova@ayu.edu.kz (K.S.); nuskabaeva.gulnaz@ayu.edu.kz (G.N.)

**Keywords:** newborn screening, severe combined immunodeficiency (SCID), X-linked agammaglobulinemia (XLA), TREC, KREC, inborn errors of immunity, dried blood spots, cut-off value

## Abstract

The early detection of Severe Combined Immunodeficiency (SCID) and X-linked agammaglobulinemia (XLA) prevents fatal outcomes. This study presents the first pilot TREC/KREC newborn screening (NBS) program in southern Kazakhstan, a high-birth-rate region, to establish local reference ranges and assess operational viability. A multiplex real-time PCR assay was used to quantify T-cell (TREC) and kappa-deleting recombination excision circles (KRECs) from dried blood spots of 5000 unselected neonates. Biomarkers were normalized to copies per $10^{6}$ cells using albumin as a diploid reference gene. Regional 0.5th percentile cut-offs were established (TREC < 3165 copies/$10^{6}\;cells$ and KREC < 2554 copies/$10^{6\;}$ cells), and gender and gestational age did not significantly impact biomarker levels. While a low birth weight (≤2500 g) significantly reduced KREC levels, the extreme lower distribution tails remained unaffected, validating the use of universal, unstratified thresholds. Applying these cut-offs yielded an optimal 1.0% initial recall rate. Consistent with global incidence rates, no true positive cases were identified. The established assay and universal percentile cut-offs demonstrate high levels of analytical reliability and demographic stability. This pilot confirms the regional pediatric healthcare infrastructure’s readiness for a routine, population-based NBS program without the need for complex algorithms.

## 1. Introduction

In the context of Inborn Errors of Immunity (IEI) care, newborn screening (NBS) for Severe Combined Immunodeficiency (SCID) using T-cell receptor excision circles (TRECs) represents one of the most successful public health interventions [1]. SCID is a life-threatening form of IEI defined by the profound dysfunction of T- and B-lymphocytes and a genetic heterogeneity involving more than 20 causal genes [2]. The only curative treatment is hematopoietic stem cell transplantation (HSCT) or gene therapy, with survival rates exceeding 92% compared to those identified by clinical illness (79.9%) or family history (85.4%) [3]. Without early detection, mortality approaches 100% [4]. The subsequent addition of kappa-deleting recombination excision circles (KRECs) is important for the detection of B-cell deficiencies, such as X-linked agammaglobulinemia. The use of the combined TREC/KREC assay on dried blood spots (DBSs) is now an established and robust method for population-based screening [5].

The successful implementation of TREC/KREC screening requires population-specific validation, as assay performance depends on the establishment of appropriate threshold cut-off values. Factors such as gestational age, birth weight, and sample conditions can influence TREC/KREC levels, necessitating large-scale data from confirmed healthy cohorts to define reference intervals. Appropriately established cut-offs must reliably distinguish between true SCID cases and healthy infants in order to minimize false positives, thereby ensuring high sensitivity for case detection and high specificity to avoid unnecessary follow-up testing [6].

**Figure 1:**
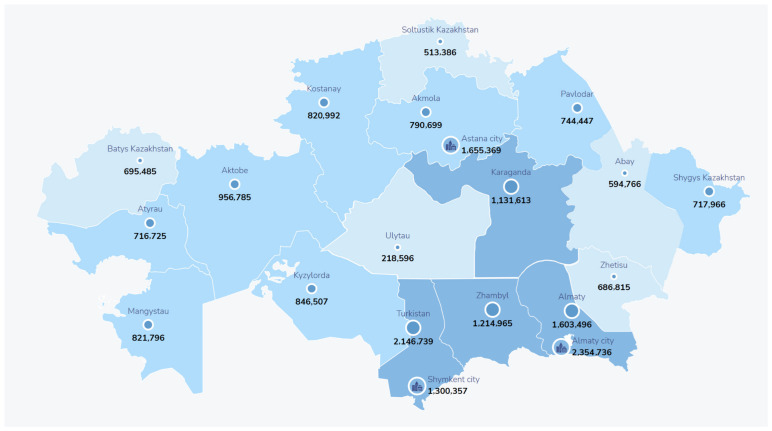
Demographic landscape and estimated annual birth cohorts across Kazakhstan. The color gradient indicates the size of the annual birth cohorts in each region, with darker shades representing higher numbers of births.

With a population of over 20 million and approximately 17.12 live births per 1000, Kazakhstan faces significant challenges related to IEI diagnosis, especially in densely populated southern regions such as Turkestan (population of 2.14 million) and Shymkent (population of 1.3 million) (Figure 1), which account for the country’s highest birth rates [7,8,9,10]. Recent national data reveal that SCID represents 5.12% of diagnosed IEI cases (out of 269) and is associated with a mortality rate of 50%. Critically, the median age for SCID diagnosis is 1.9 years, reflecting a dangerously delayed diagnostic window when early intervention would be most effective. This diagnostic delay reflects the absence of newborn screening and limited awareness among healthcare professionals, directly contributing to fatal outcomes. Furthermore, most patients present with severe vaccine-associated complications, including Bacillus Calmette–Guérin (BCG) infections (BCGosis), as the live attenuated vaccine is administered universally at birth in Kazakhstan [11,12]. While newborn screening results are typically not available in time to prevent this initial vaccine exposure (administered in the first 1–4 days of life), early detection via TREC/KREC quantification remains critical. It allows healthcare providers to immediately initiate targeted anti-microbial prophylaxis before systemic dissemination (BCG-osis) occurs, and it protects the infant from other opportunistic infections prior to definitive treatments [12].

Allogeneic hematopoietic cell transplantation (allo-HCT) remains the standard curative treatment for SCID worldwide [13]. With a timely diagnosis via NBS, HSCT can be performed in the first months of life, achieving survival rates above 92% [3]. To facilitate this, Kazakhstan’s existing national NBS program, mandated by the Ministry of Healthcare and fully state-funded since 2007, achieves 97% coverage for live births [14]. In our setting, the combination of NBS and accessible allo-HCT offers a clear pathway for the successful integration of TREC/KREC assays and the reduction of SCID mortality rates.

Despite the global implementation of TREC/KREC screening, there is no data from Central Asia, especially Kazakhstan, where genetic diversity, healthcare infrastructure, and universal BCG vaccination programs create a unique screening context. This study aims to (i) present the results of the first pilot TREC/KREC screening program in South Kazakhstan, (ii) validate the analytical robustness of our assay in this previously unreported population, (iii) establish the first population-specific threshold cut-offs and baseline TREC/KREC distributions for Kazakhstan, and (iv) demonstrate the feasibility of implementing national NBS to enable early allo-HCT and prevent life-threatening BCG complications in infants with SCID across the region.

## 2. Materials and Methods

### 2.1. Study Design and Setting

This was a prospective pilot study conducted in Kazakhstan, representing the first newborn screening initiative for SCID using TREC and KREC analyses in Central Asia. This study was designed to validate the assay performance, establish population-specific reference intervals, and assess the feasibility of implementing routine screening in Kazakhstan.

### 2.2. Study Population and Sample Selection

A total of 5000 neonates born in Turkestan and Shymkent, Kazakhstan, were enrolled between July 2025 and February 2026. Participants were chosen as a part of a consecutive, unselected cohort of live births, including premature infants, to capture a fully representative demographic spectrum and eliminate selection bias. Participation in this pilot study incurred no extra financial charge to the families. Written informed consent for both routine screening and the concurrent analysis was obtained from the parents or legal guardians of all participants.

Dried blood spot (DBS) samples were collected during the routine NBS collection on standardized Whatman 903 filter paper (Cytiva, Marlborough, MA, USA) via sterile capillary punctures. This research was strictly observational and did not deplete the biomaterial required for the mandated state screening program, nor did it cause any disruption to the routine NBS workflow. To prevent test interference, blood was collected strictly without EDTA. Samples were applied directly to the filter paper to form uniform, fully saturated circles (≥12 mm diameter). Following standard pre-analytical protocols, cards were dried horizontally for 2–3 h at room temperature (18–25 °C) and stored in airtight bags with desiccant at −20°C until analysis at the central laboratory.

In alignment with standard high-throughput NBS workflows worldwide, the primary DNA analysis was performed in a single replicate. While duplicate testing is common in initial assay development, single-replicate screening is the accepted logistical and economic standard for population-level NBS. To ensure high analytical reliability and continuously monitor intra- and inter-assay variations, standardized positive and negative quality control samples were included in every PCR plate. Demographic variables (birth date, sex, gestational age, and birth weight) were recorded for statistical analysis.

During the active sample collection phase, preliminary thresholds (TREC ≤ 2200 copies/$10^{6}\;cells$ and KREC ≤ 2000 copies/$10^{6}\;cells$) were implemented strictly as an interim clinical safety measure. Regarding clinical follow-up, any sample flagged below these preliminary values was immediately subjected to an internal laboratory repeat analysis from the original DBS. If a sample were to be confirmed as critically low (a true positive), the established protocol dictated that the newborn would be immediately referred to regional immunology and medical genetic centers for clinical follow-up and confirmatory flow cytometry, utilizing the existing government-supported diagnostic pathways. Once the complete 5000-neonate cohort was assembled, a retrospective statistical analysis was performed to calculate the definitive, population-specific 0.5th percentile cut-offs, which ultimately supersede these interim thresholds for routine regional screening.

### 2.3. Deoxyribonucleic Acid (DNA) Extraction

Genomic DNA was extracted from a single 3.2 mm DBS punch using the CAMOMILE-NkMag-PCR nucleic acid extraction kit (Diamed Asia LLC., Almaty, Kazakhstan) according to the manufacturer’s instructions. This kit utilizes magnetic bead-based technology for manual extraction. DNA was eluted in 50 µL of elution buffer and stored at −20 °C until PCR analysis.

#### Equipment and Instrumentation

All PCR reactions were performed on a CFX96™ Real-Time PCR System (Bio-Rad, Hercules, CA, USA), and DNA extraction was carried out manually. The following equipment was used according to standard laboratory protocols: a class II biological safety cabinet, a microcentrifuge, a vortex mixer, adjustable single-channel pipettes (0.5–10 µL, 10–200 µL, 100–1000 µL), and filter pipette tips free of nucleases. Sterile, nuclease-free 0.2 µL PCR tubes and 1.5 mL microcentrifuge tubes were used throughout the procedure.

Quantitative real-time PCR (qPCR) was performed using a multiplex TaqMan-based assay. Target-specific probes were labeled with distinct fluorescent reporter dyes to enable simultaneous detection in a single reaction well: FAM (6-carboxyfluorescein) was used for detection of TREC, while HEX (hexachlorofluorescein) was designated for KREC amplification. The optical separation between the emission spectra of FAM (green channel) and HEX (yellow channel) prevented signal cross-talk, ensuring high analytical specificity for both biomarkers. Amplification of an internal reference gene was routinely performed to confirm sufficient DNA yield and the absence of PCR inhibitors in the DBS extracts.

### 2.4. Multiplex Real-Time PCR for TREC, KREC, and Reference Gene

The combined TREC/KREC assay was performed using the CAMOMILE-B&T test reagent kit according to the manufacturer’s instructions. The assay simultaneously detected

- TREC as a marker of T-cell production;
- KREC as a marker of B-cell production;
- Albumin (ALB) as a reference gene for sample quality control.

### 2.5. Quality Control and Assay Validation

Each 96-well plate included the following controls as specified by the kit protocol:

- Negative control (OK): PCR-grade water instead of DNA to monitor reagent contamination. No amplification was permitted; signal detection indicated contamination requiring plate invalidation.
- Calibrators (K1–K4): four calibrators with known target concentration were used to construct the standard curve and evaluate amplification efficiency.
- Endogenous ALB control: Albumin amplification served as an internal quality control, confirming successful DNA extraction, absence of PCR inhibitors, and sufficient sample quantity. Samples in which ALB failed to amplify or exceeded acceptable Cq thresholds were considered invalid and excluded from analysis.

Amplification efficiency was assessed using the ROX channel. In cases of reduced efficiency, the lowest-concentrated calibrator (K4) could be excluded from the standard curve, as it represents the most sensitive control point. The primary validity criterion was the correct performance of all calibrators and quality controls.

Interpretation of quantification cycle (Cq) values:

- Cq values ≤ 31 cycles were considered indicative of reliable amplification.
- Cq values > 31 cycles were interpreted with caution, as they could represent extremely low DNA copy numbers, non-specific amplification, or background signal. Such samples were evaluated in conjunction with control reactions.

All DNA samples were analyzed in single replicate per the pilot study protocol.

Final result validation criteria:

A sample result was considered valid only if all of the following conditions were met:

1. Negative control demonstrated no amplification;
2. Calibrators (K1–K4, or K1–K3 if K4 was excluded) performed within acceptable parameters;
3. Endogenous ALB control was successfully amplified with Cq values within the established range;
4. Amplification curves exhibited correct sigmoidal morphology.

After confirmation of these parameters, absolute copy numbers of TREC and KREC were calculated using the standard curve derived from the calibrators.

#### Amplification Conditions

Amplification was carried out on a CFX96™ Real-Time PCR System (Bio-Rad, Hercules, CA, USA) under the following cycling conditions detailed in Table 1.

### 2.6. Quantification and Normalization

Quantitative analysis of TREC and KREC in each sample is conducted per 1,000,000 leukocytes (or peripheral blood mononuclear), taking into account the relative number of ALB copies (endogenous control) according to Formulas (1) and (2):

Formula (1):

(1) $$TREC\;copies/\;10^{6}\;cells\;=2\times \frac{1,000,000\;\times mean\;starting\;quantity\;TREC}{mean\;SQ(ALB)}$$

Formula (2):

(2) $$KREC\;copies/\;10^{6\;}\;cells\;=2\times \frac{1,000,000\;\times mean\;starting\;quantity\;KREC}{mean\;SQ(ALB)}$$

- SQ (TREC) = starting quantity (absolute copy number) of TREC calculated from the standard curve
- SQ (KREC) = starting quantity (absolute copy number) of KREC calculated from the standard curve
- SQ (ALB)—starting quantity (absolute copy number) of albumin reference gene
- 1,000,000 = factor to scale results to 1 million cells
- 2 = correction factor accounting for the diploid genome (2 copies of the albumin gene per cell)

### 2.7. Confirmatory Laboratory Workflow

To minimize unnecessary clinical recalls and parental anxiety, a standard internal confirmatory workflow was employed. Any initial DBS sample yielding TREC or KREC values below the established 0.5th percentile cut-offs was subjected to immediate repeat testing. A new 3.2 mm disk was punched from the original DBS card strictly from the center of a well-saturated blood spot to avoid edge effects and was re-analyzed. Only samples that remained abnormal after this internal repeat analysis would trigger clinical recall for a second independent DBS collection procedure or referral to a pediatric immunologist.

### 2.8. Statistical Analysis

To account for variations in DNA input and cell count, data were normalized and expressed as copies per $10^{6}$ nucleated blood cells. This normalization ensures a standardized comparison of TREC and KREC levels across the cohort. Preliminary data management was conducted in Microsoft Excel 2019 (Microsoft Corporation, Redmond, WA, USA). All inferential statistical analyses were performed using Stata BE 19.5 (StataCorp LLC., College Station, TX, USA).

As the sample size was *N* = 5000, the Shapiro–Francia test was used to evaluate the normality of the distribution of TREC and KREC copy numbers. Both variables exhibited significant deviation from normality (*p* < 0.001), characterized by positive skewness. Accordingly, non-parametric statistical methods were applied throughout the analysis. The results were considered statistically significant at a *p*-value < 0.05.

## 3. Results

### 3.1. Population Distribution and Determination of TREC and KREC Screening Cut-Offs

To establish Kazakhstan’s reference values, dried blood spot (DBS) samples from a pilot cohort of 5000 newborns in the southern region were analyzed between July 2025 and February 2026. The cohort consisted of 2660 boys and 2340 girls. The median gestational age of the cohort was 38 weeks (IQR: 36–40), and the median birth weight was 3200 g (IQR: 2690–3680). All samples yielded sufficient DNA samples for analysis, as confirmed by internal control gene amplification. TREC and KREC results—estimated by sex, birth weight, and gestational age—are summarized in Table 2. The overall median concentration was 5304 copies/$10^{6}$ cells for TREC and 4372 copies/$10^{6}$ cells for KREC. A Shapiro–Francia test confirmed that both TREC and KREC levels were not normally distributed (*p* < 0.001), demonstrating a strong positive skew. Consequently, results are reported as medians percentiles.

**Figure 2:**
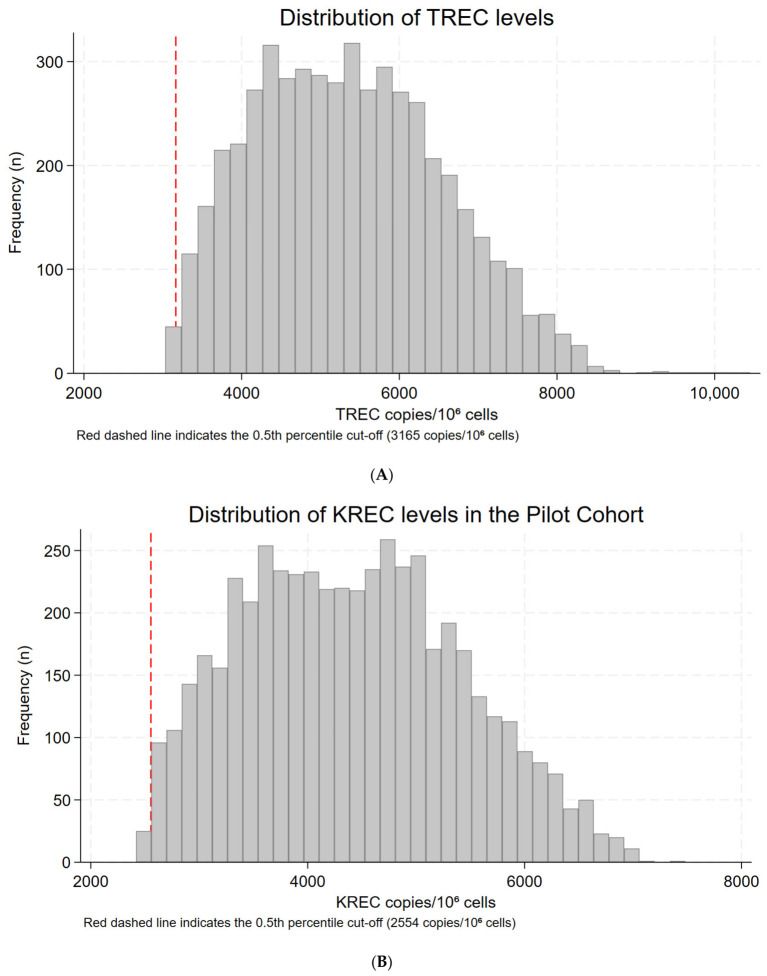
Frequency distribution of TREC and KREC levels in pilot screening cohort (*N* = 5000). Red dashed lines indicate the established 0.5th percentile cut-offs. (**A**) Distribution of TREC concentrations (cut-off: 3165 copies/$10^{6}$ cells). (**B**) Distribution of KREC concentrations (cut-off: 2554 copies/$10^{6}$ cells). Both biomarkers exhibit a pronounced non-normal, positively skewed distribution (Shapiro–Francia test, *p* < 0.001).

To ensure an adequate sensitivity for the detection of profound lymphopenia while maintaining a sustainable recall rate, the screening thresholds were established at the 0.5th percentile of the pilot cohort (*N* = 5000). Consequently, the regional cut-offs for TREC and KREC were 3165 copies/$10^{6}$ cells and 2554 copies/$10^{6}$ cells, respectively. The frequency distributions of both markers, in conjunction with the specified cut-off lines, are visualized in Figure 2, demonstrating that the selected thresholds safely isolate the extreme lower tail of the population.

#### Demographic Influences and Biomarker Distribution

**Figure 3:**
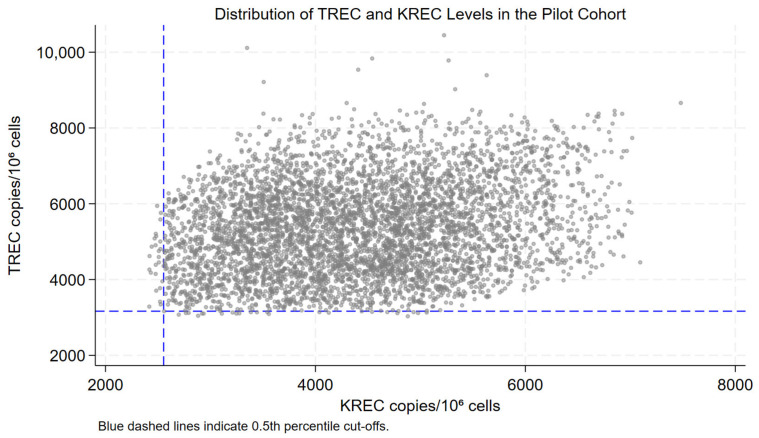
Distribution of TREC and KREC levels in the pilot cohort (*N* = 5000). The scatter plot illustrates the quantitative distribution of T-cell and B-cell excision circles. Blue dashed lines indicate the established screening cut-offs for TREC (<3165 copies/$10^{6}$ cells) and KREC (<2554 copies/$10^{6}$ cells). The absence of cases in the critical lower-left quadrant confirms that no profound T-B-SCID phenotypes were detected in this population.

The scatter plot (Figure 3) demonstrates a dense clustering of the pilot cohort within the normal physiological range, while a weak statistical correlation was observed between the markers (Spearman’s rho = 0.202, *p* < 0.001)—likely reflecting pre-analytical variables such as the overall DNA extraction yield from the dried blood spots rather than biological interdependence. TREC and KREC remain biologically independent analytes. Notably, no infants were detected in the critical lower-left quadrant (representing a simultaneously profound decrease in both T- and B-cells), visually confirming the absence of severe T-B- SCID phenotypes in this screened population.

**Figure 4:**
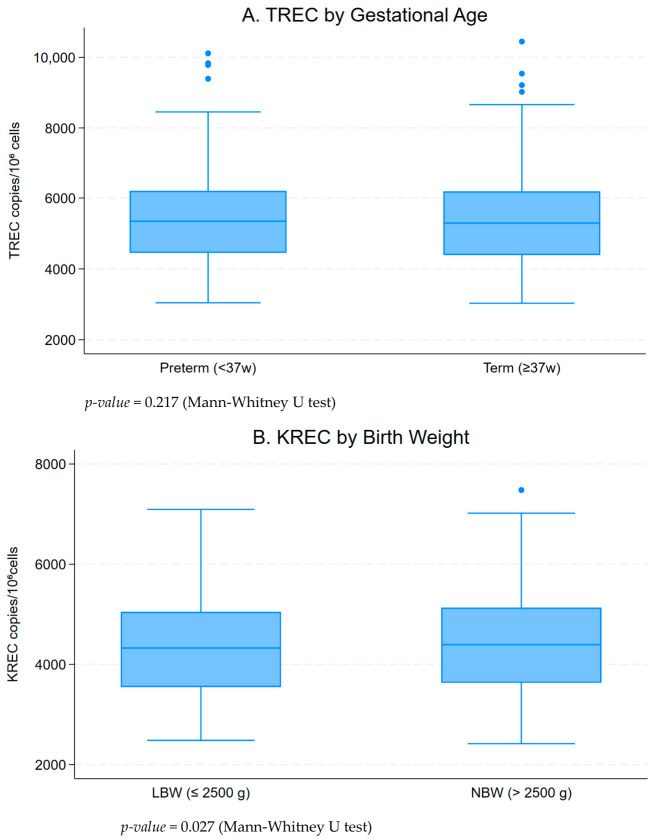
Distribution of newborn screening biomarkers stratified by key demographic factors. (**A**) TREC levels compared between preterm (<37 weeks) and term (≥37 weeks) neonates, showing no statistically significant difference (*p* = 0.217). (**B**) KREC levels compared between low-birth-weight (LBW, ≤2500 g) and normal-birth-weight (NBW, >2500 g) infants. A minor, statistically significant reduction in median KREC levels was observed in the LBW cohort (*p* = 0.027). Box plots represent the median (horizontal line) and interquartile ranges (IQRs). Comparisons were performed using the Mann–Whitney U test.

Further analysis evaluated the impact of baseline demographic factors on biomarker concentrations (Table 1). Subgroup analyses using the Mann–Whitney U test indicated that TREC and KREC levels remained remarkably stable across gender categories (*p* > 0.05). Gestational age also exhibited no significant impact on TREC levels (*p* = 0.217) when comparing preterm (<37 weeks) and term (≥37 weeks) neonates (Figure 4).

However, birth weight demographics revealed a minor but statistically significant impact on KREC concentrations (Figure 4). Infants classified as having a normal birth weight (NBW > 2500 g) exhibited slightly higher median KREC levels compared to the low-birth-weight (LBW ≤ 2500 g) cohort (*p* = 0.027). Despite this statistical significance, the interquartile ranges of both groups massively overlapped, and lower tails of the distributions remained highly comparable. This indicates that physiological variations associated with lower birth weights do not drastically shift the lower tail of the KREC distribution, confirming that universal, rather than weight-adjusted, cut-offs are sufficient for this screening program.

### 3.2. Retest Rate and False Positive Analysis

The analytical performance of the established 0.5th percentile cut-offs is detailed in Table 3. The initial screening run flagged 25 neonates (0.50%) below the TREC threshold and 25 neonates (0.50%) below the KREC threshold. Notably, cross-tabulation revealed no instances of simultaneous profound depression in both markers within the same sample. Consequently, the cumulative initial flag rate—defined as an abnormal first result requiring a secondary test from the original DBS—was 1.0% (50 out of 5000 neonates). Following the internal confirmatory laboratory workflow (repeat punching from the center of the original DBS and re-analysis), all 50 flagged samples yielded normal TREC and KREC values. These initial low readings were attributed to pre-analytical variations, such as uneven blood saturation on the filter paper or suboptimal initial punch locations. Consequently, all 50 cases were resolved as analytical false positives. Because no secondary fresh blood samples or referrals to a pediatric immunologist were required, the true clinical recall rate for this pilot phase was 0%.

Overall, the evaluation of the 5000-neonate pilot cohort successfully established regional 0.5th percentile cut-offs for TREC (3165 copies/$10^{6}$ cells) and KREC (2554 copies/$10^{6}$ cells). The demographic analysis confirmed that these universal thresholds are appropriate, as neither gender, gestational age, nor birth weight produced clinically critical shifts in the extreme lower tails of the biomarker distributions. The application of these strict cut-offs, supported by robust assay precision, resulted in a highly manageable initial recall rate of 0% with zero true positive cases of severe immunodeficiency. Together, these findings demonstrate that the established screening protocol is both analytically reliable and logistically sustainable for the southern region’s pediatric healthcare infrastructure.

## 4. Discussion

### 4.1. Principal Findings and Real-World Viability

This study presents the successful implementation of a pilot newborn screening program for Severe Combined Immunodeficiency (SCID) and X-linked agammaglobulinemia (XLA) in 5000 neonates in South Kazakhstan. Our findings demonstrate the robust analytical performance and practical viability of using a multiplex real-time PCR assay for the simultaneous quantification of TREC and KREC from dried blood spots. The most significant achievement of this pilot phase was the establishment of regional, population-based 0.5th percentile cut-offs (TREC < 3165 and KREC < 2554 copies/$10^{6}$ cells), which resulted in a precise initial laboratory retest rate of exactly 1.0% and a clinical recall rate of 0%. By achieving an optimal, highly manageable initial laboratory retest rate, this protocol ensures rigorous screening safety without overburdening the regional pediatric healthcare infrastructure, validating its readiness for routine, large-scale clinical application.

### 4.2. International Comparison and Assay Methodology

When evaluating our regional cut-offs against international data, it is imperative to account for fundamental methodological differences in assay calibration. Currently, there is no universally adopted consensus for SCID screening thresholds, leading to variable TREC and KREC assay protocols in laboratories worldwide. Therefore, given the inherent methodological variations in TREC/KREC assays and the recognized impact of population genetics on biomarker distributions, substantial variations exists across different screening programs (Table 4). In this pilot study, TREC and KREC concentrations were quantified as copies per $10^{6}$ cells using albumin as a diploid reference gene, rather than relying on volume-based quantification (copies/µL of blood). A direct numerical comparison with volume-based thresholds from other programs is mathematically invalid.

### 4.3. Demographic Stability and the Clinical Relevance of Cut-Offs

For national newborn screening programs, a major operational challenge is the potential need for complex, stratified diagnostic algorithms based on patient demographics. However, our subgroup analysis revealed that both TREC and KREC levels remained remarkably stable across gender categories (*p* > 0.05). While a study conducted in Hong Kong indicates that gender-based disparities in KREC levels arise in late childhood [23], our data confirms that such stratification is unnecessary during the neonatal period. This aligns with the operational frameworks of established European programs, such as the Swiss newborn screening initiative, which use universal thresholds without gender-specific data [24].

Similarly, while some international studies, such as Kinoshita Y. et al., indicate that prematurity and low birth weights can reduce TREC and KREC concentrations [25], our data identified no significant impact of gestational age on TREC (*p* = 0.217). More importantly, any minor physiological shifts related to birth weight did not affect the 0.5th percentile threshold. This robust demographic stability solidifies the validity of using simple, universal screening cut-offs for the entire regional population.

According to the study by Cheremokhin et al., TREC levels during fetal growth increase, but there is no significant correlation between preterm categories and TREC levels. Furthermore, they did not report a significant relationship between KREC and newborn weight or gestational age parameters, concluding that neonates do not need to be divided into separate diagnostic groups based on these factors [26]. This robust demographic stability solidifies the validity of using simple, universal screening cut-offs for the entire regional population.

### 4.4. Assay Reliability and Incidence Expectations

The assay’s analytical reliability is demonstrated by the dense clustering of the cohort within the normal physiological range (Figure 2). Although TREC and KREC are biologically independent analytes, and SCID is present in various phenotypes, the absence of cases in the critical lower-left quadrant confirms that no severe T-B-SCID or profound generalized lymphopenia cases were present, highlighting the cohort’s healthy immunological baseline.

No true positive cases of SCID or XLA were detected among the 5000 neonates. Given the global SCID incidence of 1 in 50,000 to 100,000 live births, this cohort size is mathematically insufficient to identify a true positive case. However, establishing the disease prevalence rate was not the primary goal; this pilot aimed to verify the assay’s analytical characteristics. The highly manageable initial laboratory retest rate of 1.0% and the 0% clinical recall rate confirm that the regional infrastructure is fully prepared for large-scale implementation. The program will inevitably detect true positive cases and enable early, life-saving interventions. Crucially, it should be noted that in Kazakhstan, the routine BCG vaccine is administered during the first 1–4 days of life, which generally precedes the availability of NBS results. Therefore, while newborn screening cannot prevent initial infant exposure to this live attenuated vaccine, the early identification of severe lymphopenia is critically vital. This enables healthcare providers to immediately initiate targeted anti-mycobacterial prophylaxis therapy, significantly reducing the risk of fatal disseminated BCG-osis, and facilitates early protection against other opportunistic infections while the patient is awaiting definitive therapy.

### 4.5. Strengths and Limitations

We acknowledge two primary limitations. First, the sample size was strictly capped at 5000 neonates due to the financial constraints of the pilot grant. While this cohort size is statistically robust for establishing baseline reference percentiles, it yielded no true positive cases, limiting our ability to prospectively assess the clinical sensitivity and positive predictive value (PPV). Second, we used single-replicate primary screening. Although this is the universally accepted economic and logistical standard for high-throughput NBS, this method relies on continuous plate-level quality controls to monitor intra- and inter-assay variations.

Despite these constraints, a major strength of this study is the use of a comprehensive, unselected cohort that perfectly represents the regional demographic. Because population genetics and specific assay methodologies influence biomarker distributions, relying on imported international cut-offs is clinically inadequate. Therefore, these locally validated thresholds provide a highly accurate, evidence-based foundation for routine NBS implementation in South Kazakhstan.

## 5. Conclusions

In conclusion, this pilot study successfully established and validated local TREC and KREC screening thresholds for the southern region of Kazakhstan. The multiplex PCR assay demonstrated high analytical precision, and the strict cut-offs resulted in a highly efficient 0% clinical recall rate. These findings provide strong evidence-based support for the integration of TREC/KREC quantification into the routine national newborn screening panel, which leads to the early detection and management of Severe Combined Immunodeficiency in our population.

## Figures and Tables

**Table 1:** Cycling conditions.

Stage	Step	Temperature	Time	Number of Cycles
1	Initial denaturation	95 °C	3 min	1
2	Denaturation	95 °C	10 s	5
	Primer annealing	60 °C	30 s	
	Extension	72 °C	15 s	
3	Denaturation	95 °C	10 s	40
	Primer annealing/signal acquisition	60 °C	30 s (fluorescence reading)	
	Extension	72 °C	15 s	

**Table 2:** Demographic data and TREC and KREC values for the population.

	Data	Number	%	TREC Copies/$10^{6}\;Cells,$ Median (IQR)	*p*-Value	KREC Copies/$10^{6}\;Cells,$ Median (IQR)	*p*-Value
Gender	Male	2660	53.2	5294 (4413–6208)	0.609	4350 (3598–5099)	0.205
	Female	2340	46.8	5306 (4416–6183)		4397 (3628–5142)	
Gestational age	Born at term, ≥37 weeks	3580	71.6	5291 (4391–6188)	0.217	4357 (3622–5097)	0.656
	Preterm, <37 weeks	1420	28.4	5345 (4453–6202)		4412 (3590–5167)	
Weight at birth	NBW > 2500 g	3811	76.22	5295 (4416–6203)	0.999	4394 (3632–5133)	0.027
	LBW ≤ 2500 g	1189	23.78	5327 (4400–6176)		4327 (3548–5049)	
Overall samples		5000	100	5304 (4415–6194)	*N/A*	4372 (3614–5116)	*N/A*

The Kruskal–Wallis test and the Mann–Whitney U test were used for the comparative analysis between groups (*p*-values were determined at a significance threshold of 5%); IQR—interquartile range; *N/A*—not applicable; LBW—low birth weight; NBW—normal birth weight.

**Table 3:** Analytical performance and initial recall rates of the TREC/KREC screening assay.

Biomarker	Cut-Off (0.5th Percentile)	Number of Screen Flags	Initial Laboratory Retest Rate (%)	Confirmed SCID/XLA Cases
TREC only	<3165 copies/$10^{6}$ cells	25	0.50%	0
KREC only	<2554 copies/$10^{6}$ cells	25	0.50%	0
Both TREC and KREC	Below both thresholds	0	0.00%	0
Total	-	50	1.00%	0

**Table 4:** Variability in TREC and KREC screening thresholds among selected national cohorts.

Study	Assay Type/Methodology	TREC Cut-Off	KREC Cut-Off	Unit of Measurement
This study	Multiplex real-time PCR assays	<3165	<2554	copies/$10^{6}$ cells
Gutiérrez-Hincapié S. et al. (Colombia, 2024) [15]	Multiplex TaqMan qPCR	119	69	copies/pl of blood
Chen C. et al. (China, 2024) [16]	Multiplex real-time PCR assays (NeoMDx PCR kit, Xinbo, Suzhou, China)	< 399	<124	copies/$10^{5}$ cells
Boyarchuk O. et al. (Ukraine, 2025) [17]	RT-qPCR (TRECs/KRECs/ACTB-assay)	<5000	<2000	copies/per $10^{6}\;$ cells
Marinova M. et al. (Bulgaria, 2022) [6]	Commercial EnLite Neonatal TREC kit (Wallac Oy, Mustionkatu 6, FI-20750 Turku, Finland)	≥36	≥13	copies/µL
Gutierrez-Mateo C. et al. (Denmark, 2019) [18]	Multiplex real-time PCR assays (NeoMDx PCR kit, PerkinElmer, Turku, Finland)	380	231	copies/$10^{5}$ cells
Barbaro M. et al. (Sweden, 2016) [19]	Quantitative triplex real-time qPCR for TREC, KREC, and beta-actin (ACTB)	≤15	≤10	copies/3.2 mm blood spot
de Felipe B. et al. (Spain, 2015) [20]	Triplex RT-PCR	6	4	/punch
Kwan A. et al. (USA, 2014) [21]	RT-qPCR	heterogeneous profile (11 states)	-	various
Borte S. et al. (Sweden, 2012) [22]	Quantitative triplex real-time qPCR for TREC, KREC	15	10	µL

## Data Availability

The data are not publicly available due to ethical and privacy restrictions. All relevant statistical analysis and reference percentiles supporting the findings of this study are included in the manuscript.

## Abbreviations

The following abbreviations are used in this manuscript:

IEI	Inborn errors of immunity
SCID	Severe combined immunodeficiency
XLA	X-linked agammaglobulinemia
NBS	Newborn screening
DBS	Dried blood spot
TREC	T-cell receptor excision circle
KREC	Kappa-deleting recombination excision circle
HSCT	Hematopoietic stem cell transplantation
Allo-HCT	Allogeneic hematopoietic cell transplantation
ALB	Albumin
LBW	Low birth weight
NBW	Normal birth weight
SQ	Starting quantity
RT-qPCR	Quantitative real-time PCR
IQR	Interquartile range
Cq	Quantification cycle
FAM	6-carboxyfluorescein
HEX	Hexachlorofluorescein
DNA	Deoxyribonucleic acid
